# A Successful Method of Treating Certain Cases of Dysmenorrhœa and Sterility

**Published:** 1871-09

**Authors:** Protheroe Smith

**Affiliations:** Senior Physician to the Hospital for Women, London


### A Successful Method of Treating Certain Cases of Dysmenorhoea and Sterility.

By Protheroe Smith, M. D., Senior Physician to the Hospital for Women, London.

After giving the pathology of the obstructive dysmenorrhoea with the usual mode of treatment, Dr. Protheroe Smith called attention to that of dilation by bougies, suggested by Dr. Mackintosh, and the modification of it by Dr. Simpson, and his operation of incision of the cervix by the hysterotome. Dr. Pretheroe Smith's experience having suggested a doubt of the advantage of this practice, he was led to adopt the plan he now advocated in certain cases indicated by conditions which he mentions. To overcome the stricture of the os internum, he used *extension force* by means of his uterine dilator, an instrnment described and exhibited, as being particularly suitable to this purpose. To restore the os tincæ to its natural form, per speculum, he incised it laterally at the commissures of the labia. Particular instructions were given for this treatment, which, during the last twenty-five years, he had employed with considerable success in remedying both obstructive dysmenorrhoea and sterility. Six cases were cited in illustration.—*British Medical Journal.*

